# Nano-Pulse Stimulation Therapy in Oncology

**DOI:** 10.1089/bioe.2024.0009

**Published:** 2024-06-12

**Authors:** Richard Nuccitelli, Amanda McDaniel

**Affiliations:** Pulse Biosciences, Inc., Hayward, California, USA.

**Keywords:** nano-pulse stimulation, nanosecond, nanosecond pulsed electric fields, regulated cell death, cryoablation, radiofrequency ablation

## Abstract

**Background:**

Nano-Pulse Stimulation (NPS) therapy applies electric pulses in the nanosecond domain to initiate regulated cell death in the treated tissues. This nonthermal therapy has been used to treat a wide range of murine tumors and has been shown to activate the immune system to inhibit the growth of rechallenge tumors, as well as untreated, abscopal tumors when accompanied by the injection of immune system stimulants into the treated tumors. Clinical trials have begun using NPS to treat basal cell carcinoma and hepatocellular carcinoma.

**Methods:**

Murine tumors can be easily imaged when the tumor cells are injected intradermally so that they grow within the mouse skin. Pulling the skin over a translucent light post shines light through the skin and makes it easy to treat the tumor and identify the treatment zone.

**Results:**

Original research using murine tumor models is described, including melanoma, squamous cell carcinoma, lung carcinoma, breast carcinoma, and pancreatic carcinoma. The energy required to ablate these tumors has been determined with pancreatic carcinoma and lung carcinoma exhibiting 90% ablation with 240 mJ/mm^3^, lung carcinoma and squamous cell carcinoma requiring 360 mJ/mm^3^, and melanoma requiring 480 mJ/mm^3^. NPS therapy initiated a variable immune response indicated by the rejection of injected rechallenge tumor cells with melanoma and hepatocellular carcinoma exhibiting the strongest response and lung carcinoma, the weakest response. Following the original research data, a review of human clinical trials using NPS therapy is presented.

**Conclusions:**

NPS therapy offers a nonthermal, drug-free approach for oncology, which is limited only by applying energy to the tumor. This new immunogenic modality is just beginning to be applied in the clinic. The 87% efficacy of the first large clinical trial conducted by several medical personnel is impressive and indicates that NPS is an effective new modality for cancer treatment.

## Introduction

The standard of care for the treatment of most tumor types remains surgical resection followed by radiation and chemotherapy. However, if the tumor’s location can be accurately determined with some form of imaging, various other ablation modalities can be applied to treat it. These include radiofrequency ablation (RFA), cryoablation, irreversible electroporation (IRE), electrochemotherapy, and most recently, nanoelectroablation, which applies short electric pulses in the nanosecond range to trigger regulated cell death. Nanoelectroablation (most recently referred to as Nano-Pulse Stimulation™ therapy (NPS™) or Nanosecond Pulsed Field Ablation (nsPFA^TM^)) was discovered 25 years ago^1,2^ and since then has been studied by well over 1000 scientists in laboratories worldwide. However, although NPS has been found to be very effective in treating murine tumors, the application to human cancer therapy has been slow, with the first small clinical trial not occurring until 2014^3^ and the first large clinical trial not appearing until 2022.^4^ We have learned a great deal about the unique mechanisms used by NPS to initiate regulated cell death (RCD), as well as its ability to stimulate the immune system to attack the tumor.^5,6^ This could offer a novel approach to treating cancer. The mechanisms by which NPS initiates RCD have been described in detail previously.^7^ Briefly, these ultrashort electric pulses generate nanopores in both the plasma membrane and organelle membranes of treated tissues, increasing intracellular calcium, initiating DNA fragmentation and protein hydrolysis, and triggering the RCD pathway utilized by all cells when they reach the end of their useful life.^8^

It is this initiation of RCD that is the unique characteristic of NPS therapy, which will slowly eliminate treated cells and provide time for the recruitment of the immune system to remove the dying cells and initiate an immune response if the dying cells contain foreign antigens.

Another important characteristic of NPS therapy is that its effect is highly localized to the region between the bipolar applicator electrodes. As no energy is applied outside of that region, this therapy has very few side effects. Over 5000 NPS treatments to remove skin lesions have been completed to date, with the only side effect being a transient hyperpigmentation in the treated area in some subjects.^9–11^

The first applications of NPS therapy were on bacterial decontamination^12^ and inhibiting murine tumor growth.^2,13^ Since those early days, the scientific community studying the effects of nanosecond pulses on biological systems has grown to include laboratories in most countries in Western Europe, China, Japan, and Korea. These laboratories have produced over 200 publications describing NPS treatments on a wide range of murine tumor types with generally high efficacy. The main challenge is exposing every tumor cell to the NPS energy, which can be difficult when tumors are located deep in the body. In this study, we solve that problem by localizing the tumors to the skin, which is easily treated. We will first describe our original studies ablating murine tumors with NPS therapy, followed by a description of the oncology studies from other laboratories using NPS treatments.

## Materials and Methods

### Mice

Female C57BL/6J mice, 6–8 weeks old (Jackson Laboratories, Sacramento, CA), were acclimated for at least 3 days before treatment, housed in groups of 10, and both flanks were shaved before the start of tumor inoculations. Animals were maintained on a 12-h light/dark cycle. Water (Milli-Q) and food (Pirolab Diet 20 chow) were given *ad libitum*. All experiments were performed in accordance with animal care guidelines set forth by the Pulse Biosciences IACUC.

### Cell lines

Pan02 cells were obtained from the National Institutes of Health (NIH), and C3.43 cells were an *in vivo* passaged derivative of the C3 HPV16-transformed B6 murine tumor cell line.^14^ All other cell lines used came from the American Type Culture Collection (ATCC). These included 4T1 breast cancer cells, SCCVII squamous cell carcinoma cells, B16 melanoma cells, Lewis lung carcinoma cells (LLC), and McA-RH7777 rat liver tumor cells. All cell lines were propagated in tissue culture with DMEM supplemented with 10% v/v fetal bovine serum and penicillin/streptomycin and harvested for inoculation between passages 9 and 12 for all studies.

### Tumors

**FIG. 1.**
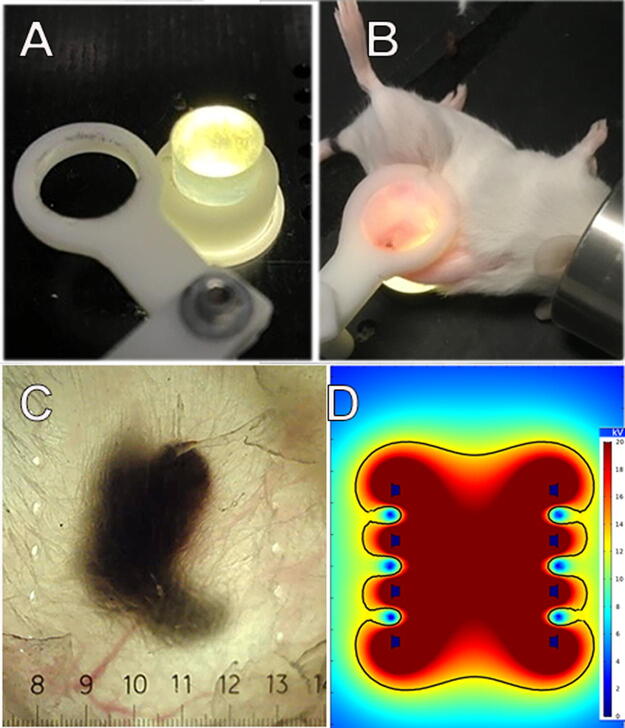
Previously unpublished method for treating the entire murine tumor. **(A)** Photo of lighted silicone post over which the skin encapsulating the tumor is stretched. The white ring is placed over the skin to hold it once the tumor is in position. **(B)** Photo of mouse with skin stretched over the post for transillumination; **(C)** Image of a melanoma tumor after treatment with two rows of bipolar needle electrodes. Holes indicate the position of the two rows of electrodes. **(D)** COMSOL Multiphysics model of the electric field distribution between the two rows of needle electrodes (black dots) indicating the uniformity of the applied electric field.

Primary tumors were initiated in mice by intradermal injection into the right flank with 1 × 10^6^ cells/30 μL in Hank’s balanced salt solution, while the mice were under isoflurane anesthesia. One way to be sure that every tumor cell is exposed to the applied field is to inject the tumor cells into mouse skin intradermally so that the tumor grows within the skin layer and can be imaged externally. We have developed such a method in which the skin containing the tumor can be stretched over a translucent silicon light post, which shines light through the skin so that the tumor can be visualized on a video screen for accurate placement of the applicator (Fig. 1). After treatment, the holes in the skin provide an accurate map of the precise region to which energy was applied (Fig. 1C). NPS therapy was delivered using a 5.0 × 5.0 × 3.5 mm treatment tip attached to a handpiece plugged into the CellFX^®^ pulse generator. The treatment tip contained two parallel rows of 5 microneedles 3.5 mm in length, and the rows were spaced 5 mm apart (Fig. 1).

### NPS treatments

Most murine tumor experiments treated a single tumor in each of 10 mice using nanosecond pulses that were 30 kV/cm in amplitude, and the change in tumor size was then recorded over time. Most tumors were completely ablated within 35 days, and the percentage of ablation was calculated as the number of mice tumor-free at that time divided by the total number treated. All statistical analyses were performed on GraphPad Prism (GraphPad Software, Inc., San Diego, CA).

## Results

**FIG. 2.**
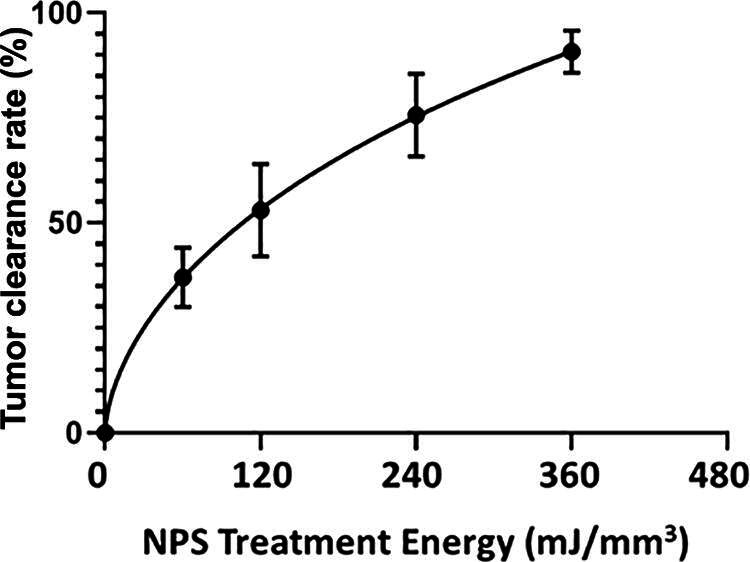
B16 murine melanoma clearance following the application of the indicated treatment energy with a bipolar applicator (error bars represent SEM.) (taken with permission from McDaniel et al.^15^).

**FIG. 3.**
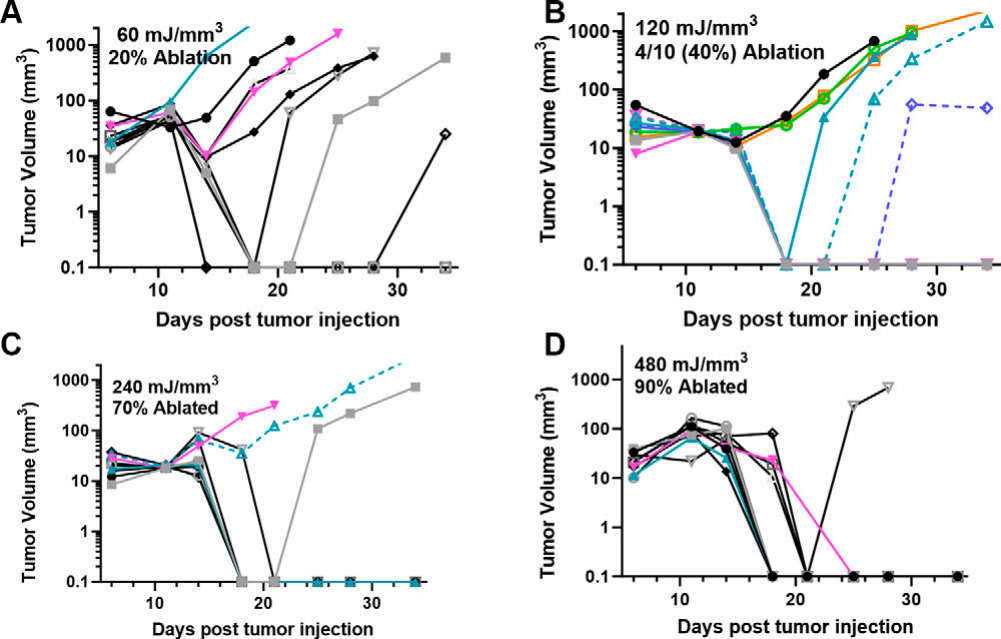
Previously unpublished growth of B16 melanoma tumors injected on day 0 and treated on day 5 with 30 kV/cm pulses with the indicated NPS energy. **(A)** 60 mJ/mm^3^ treatment ablated 2 of the 10 tumors treated; **(B)** 120 mJ/mm^3^ ablated 4 of the 10 tumors treated; **(C)** 240 mJ/mm^3^ ablated 7 of the 10 tumors treated; **(D)** 480 mJ/mm^3^ ablated 9 of the 10 tumors treated. NPS, Nano-Pulse Stimulation.

**FIG. 4.**
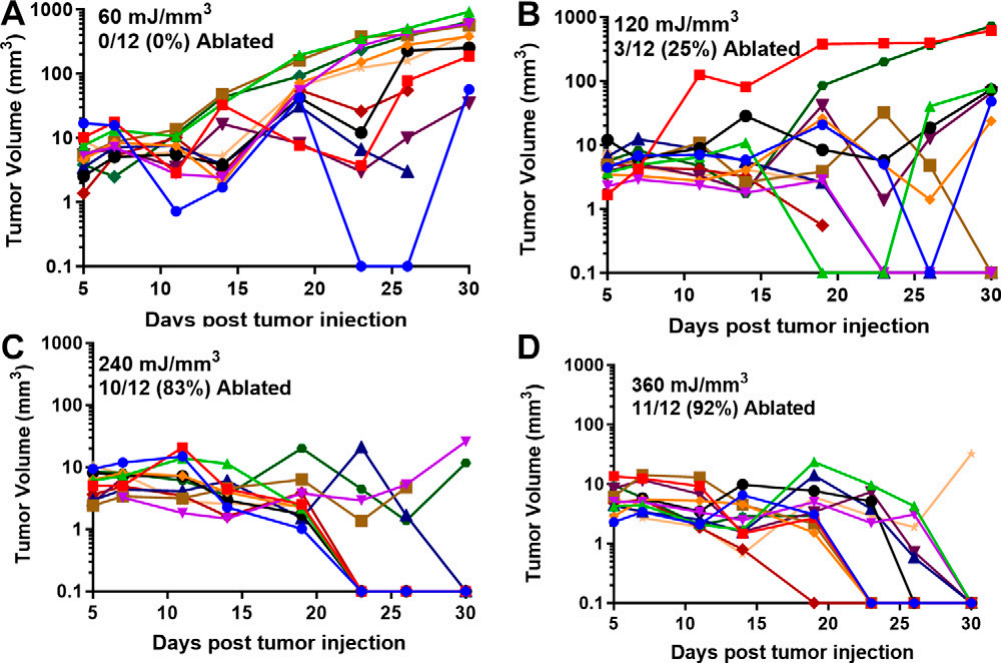
Previously unpublished growth of 4T1 breast tumors injected on day 0 and treated on day 5 with the indicated NPS energy. **(A)** 60 mJ/mm^3^ treatment did not ablate any tumors; **(B)** 120 mJ/mm^3^ ablated 3 of the 12 treated tumors; **(C)** 240 mJ/mm^3^ ablated 10 of the 12 treated tumors; **(D)** 360 mJ/mm^3^ ablated 11 of the 12 treated tumors.

**FIG. 5.**
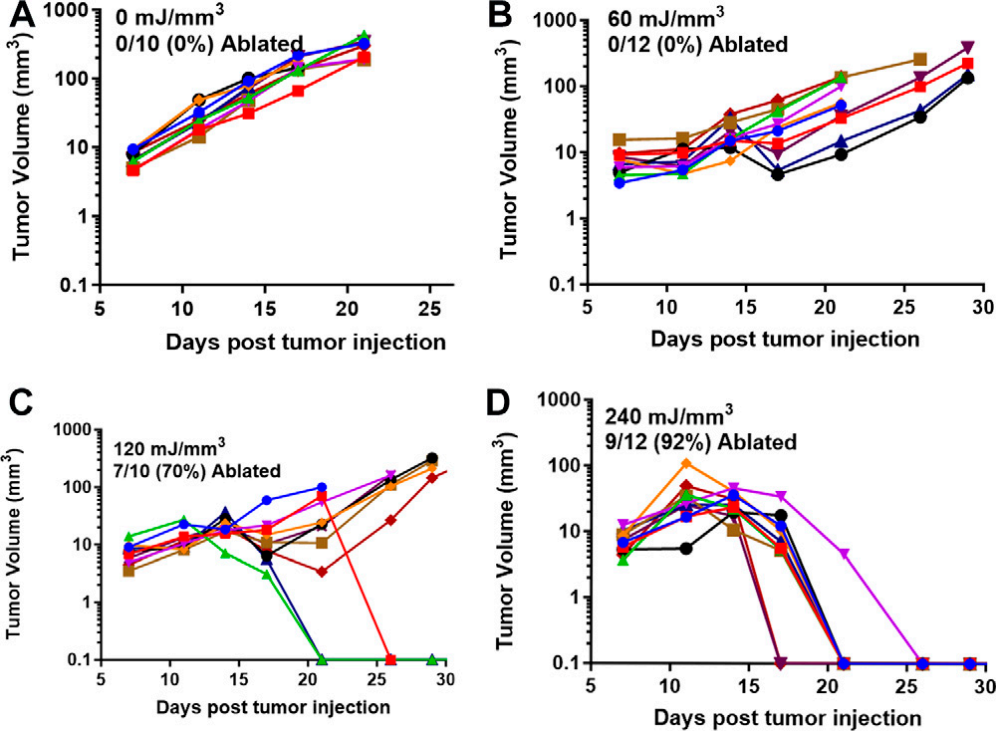
Previously unpublished growth of Lewis lung carcinomas (LLC) injected on day 0 and treated on day 5 with the indicated NPS energy. **(A)** In the sham control, none of the 10 treated tumors was ablated; **(B)** 60 mJ/mm^3^ treatment ablates none of the 12 treated tumors; **(C)** 120 mJ/mm^3^ ablates three of the 10 tumors treated; **(D)** 240 mJ/mm^3^ treatment ablated all 10 of the 10 treated tumors.

**FIG. 6.**
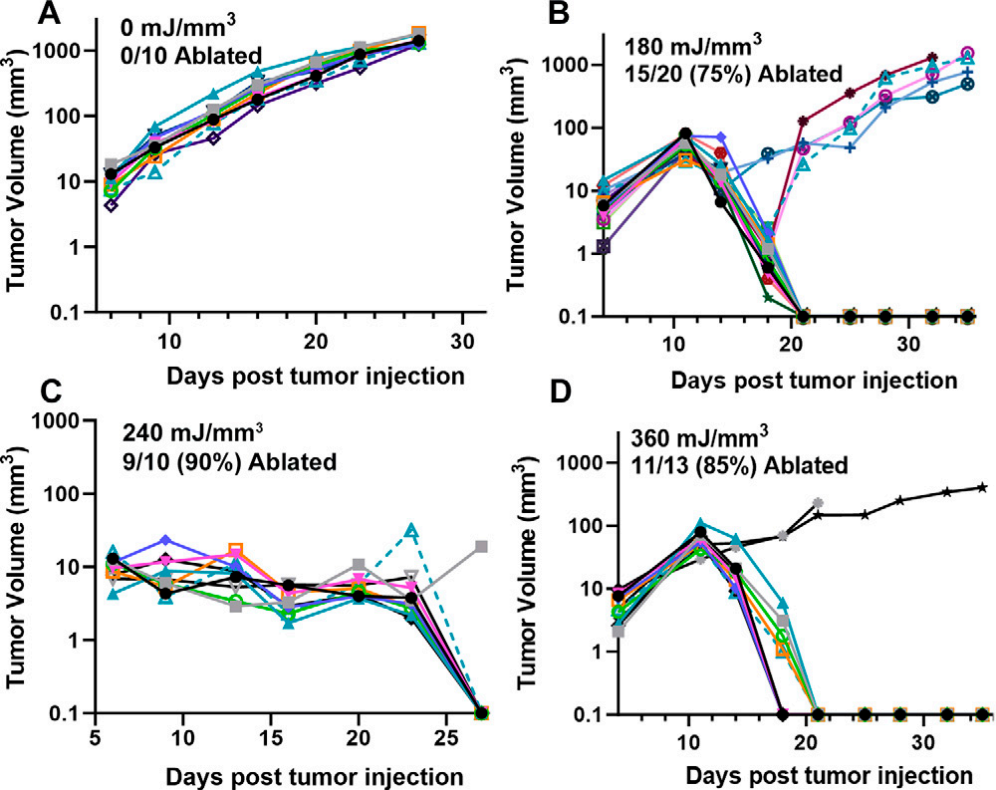
Previously unpublished growth of squamous cell carcinomas (SCCVII) injected on day 0 and treated on day 5 with the indicated NPS energy. **(A)** Untreated tumors all grow well with no ablation; **(B)** 180 mJ/mm^3^ treatment ablated 15 of the 20 treated tumors; **(C)** 240 mJ/mm^3^ treatment ablated 9 of the 10 treated tumors; **(D)** 360 mJ/mm^3^ treatment ablated 11 of the 13 tumors treated. We suspect that these two were not completely covered by the NPS treatment, resulting in the continued growth.

**FIG. 7.**
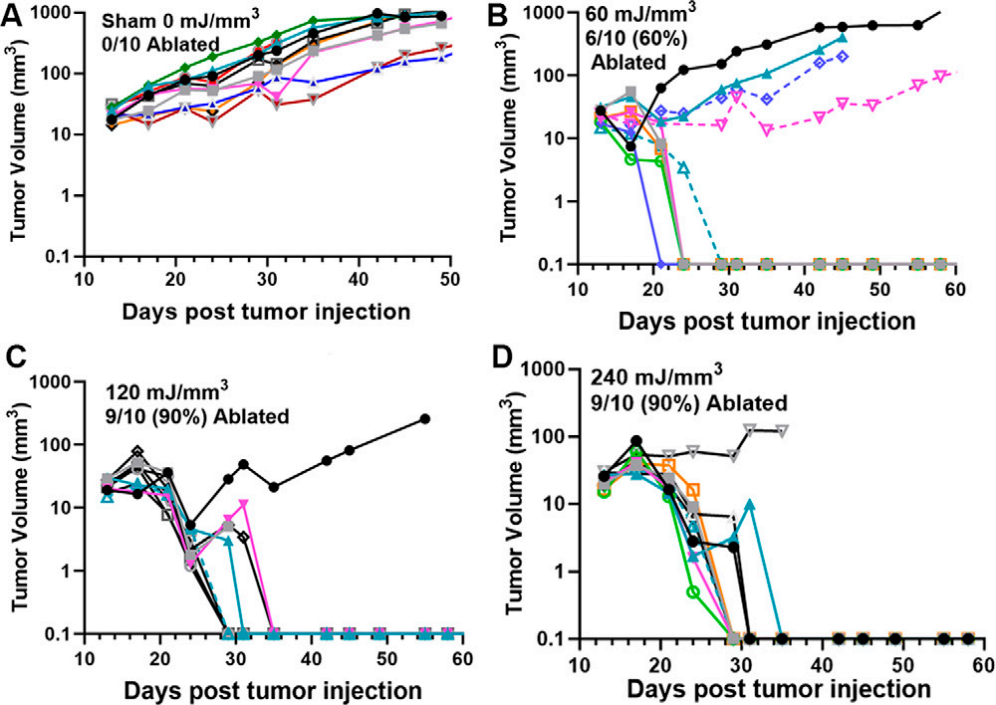
Previously unpublished growth of pancreatic (Pan02) tumors injected on day 0 and treated with NPS on day 11. **(A)** Sham tumor growth with electrodes inserted around tumor without delivering energy; **(B)** 60 mJ/mm^3^ treatment ablated 6 of the 10 tumors treated; **(C)** 120 mJ/mm^3^ treatment ablated 9 of the 10 tumors treated; **(D)** 240 mJ/mm^3^ treatment ablated 9 of the 10 tumors treated.

We have used this technique of localizing tumors in the skin to treat several tumor types and determine the energy required to eliminate them *in vivo*. One major advantage of NPS therapy is that it is not cell type-specific, and the appropriate energy will trigger regulated cell death in any cell type treated. However, the energy required to initiate cell death does vary with tumor type. Treatments that apply 1–10 mJ/mm^3^ will have very little effect on cells; 60 mJ/mm^3^ ablated 20% of the treated murine B16 melanoma tumors, but 480 mJ/mm^3^ was required to ablate 90% of the B16 tumors treated (Figs. 2, 3). In contrast, 4T1 breast cancer tumors and LLCs require twice that energy to begin exhibiting ablation (Figs. 4, 5), whereas pancreatic Pan02 tumors and squamous cell carcinomas can be ablated with lower energies than B16 melanoma tumors (Figs. 6, 7, Table 1). The main point here is that NPS therapy is extremely effective at ablating tumors when the energy can be delivered to every cell in the tumor. That means the challenge we face is energy delivery to tumors found in organs deep in the body. If the tumors can be imaged with high resolution, effective treatment will rely upon accurate positioning of the applicator electrodes to encompass the entire tumor and deliver energy uniformly unless the immune system can be stimulated to go after untreated tumor cells as explained next.

**Table 1.** Ablation Energies for the Treated Tumor Types

Tumor Type	Energy for 90% Ablation (mJ/mm^3^)
Pancreatic carcinoma	240
Lung carcinoma	240
Breast carcinoma	360
Squamous cell carcinoma	360
Melanoma	480

### Evidence for the stimulation of an immune response by NPS therapy

One of the hallmarks of regulated cell death is the release of danger-associated molecular patterns (DAMPs) that are detected by the immune system. These factors attract dendritic cells to the treated region, and the translocation of calreticulin from the endoplasmic reticulum to the NPS-treated cell surface stimulates dendritic cells to phagocytose the treated cells. This not only removes the dead cells but also enables the dendritic cells to present any non-self or foreign antigens found in the treated region to the immune system to generate an immune response such as antibodies and cytotoxic CD8+ T cells specific for the detected antigens. Evidence from our work that NPS therapy activates such an immune response^5,16^ comes largely from injecting a rechallenge tumor after the primary tumor has been ablated by NPS therapy.

### Tumor rechallenge experiments

**FIG. 8.**
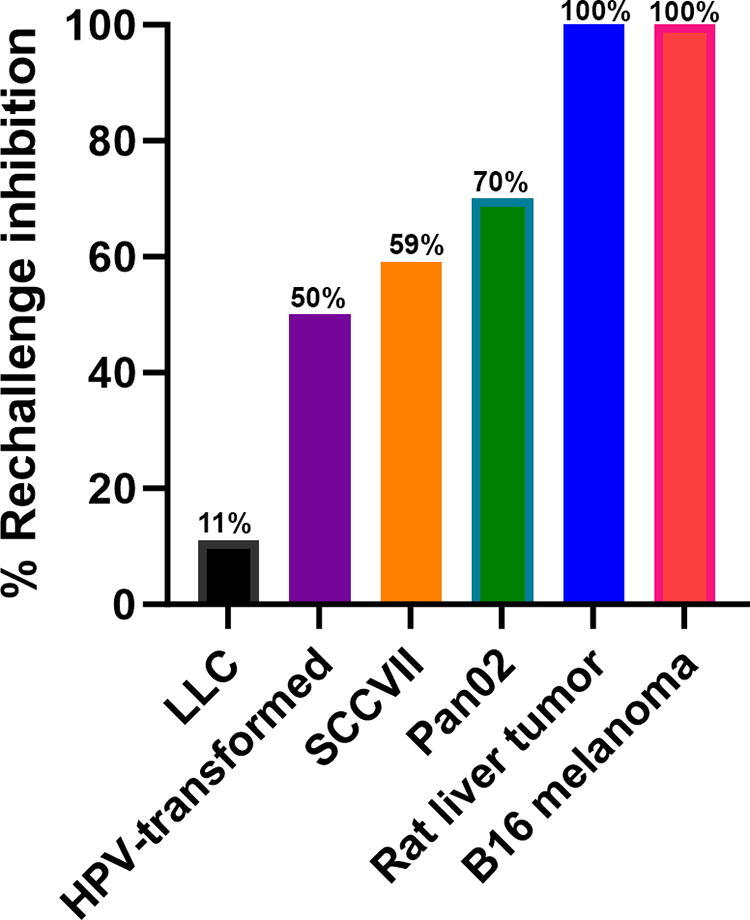
The percentage of rechallenge tumors that were inhibited in six different tumor types, indicating varying degrees of immune stimulation (previously unpublished).

A common way to demonstrate that tumor-specific CD8+ T cells have been generated is to demonstrate that a bolus of tumor cells injected at least 2 weeks after the primary tumor has been treated fails to grow into a tumor due to CD8+ T cell infiltration. We have conducted such a rechallenge experiment for several different tumor types, including murine melanomas,^17^ rat liver tumors,^18,19^ papilloma virus-transformed tumors,^14^ murine breast tumors,^20^ and pancreatic tumors,^21^ as summarized in Figure 8. The percentage of inhibition varied from 11% in LLC tumors to 100% in rat liver tumors and B16 melanoma tumors, indicating the range of immune response stimulation by NPS treatment. For the rat liver tumors and HPV-transformed tumors, we demonstrated the dependence of this inhibition on the presence of CD8+ T cells. In addition to this inhibition of tumor growth, within the NPS-treated rat liver tumors, other investigators have found that immunosuppressive T regulatory cells were reduced and the number of natural killer cells increased.^22^

In our recent work treating murine pancreatic cancer, we used immunohistochemistry to label sections of rechallenge tumors with anti-CD8 antibodies and showed that the concentration of CD8+ cells was much higher in tumors injected into mice whose primary tumor was treated with NPS than in those in which the primary tumor had been removed surgically.^21^ This provides additional evidence that NPS treatment stimulates an antitumor immune response.

## Discussion

In addition to these rechallenge experiments, studies of NPS-treated tumors exhibit changes in their immune profiles, which indicate the stimulation of an immune response. In treated Pan02 tumors, the number of CD8+ T cells increased, and there was a decrease in immune suppressive cells, including myeloid-derived suppressor cells, T regulatory cells, and tumor-associated macrophages.^16^ In NPS-treated murine liver tumors, CD103+ dendritic cells accumulate in the tumor, as did CD8+ T cells expressing interferon-gamma (IFN-γ).^23^ In the 4T1 breast cancer model, central memory CD8+ T cells greatly increased and immune-suppressing MDSC levels fell in the spleen of mice whose tumor was ablated with NPS.^20^

### Elimination of untreated abscopal tumors

Although the inhibition of rechallenge tumor growth suggests that CD8+ T cells were present, the ultimate goal is to stimulate the immune system to a degree that eliminates tumor cells outside of the immediate ablation zone. This includes parts of the tumor that were missed by the applicator or primary tumor cells that had migrated to other locations in the body. We have made some progress toward that goal in treating murine pancreatic cancer by combining NPS treatment with a T cell co-stimulatory molecule, anti-OX40 (aOX40). aOX40 has been shown to activate and expand antigen-specific tumor-infiltrating T cells.^24^ We decided to inject two tumors and treat only one so that we could monitor the growth of the untreated tumor following the clearance of the treated tumor. The regression of a distant, untreated tumor after treating the primary tumor is known as the “abscopal effect”. When we injected aOX40 intratumorally immediately after NPS treatment of the pancreatic tumor, the untreated abscopal tumors were eliminated in 80% of the mice. This indicates that the immune system could be stimulated sufficiently to attack an untreated tumor present in the same animal.

### Applicator developments

Pulse Biosciences, Inc., is developing applicators designed to deliver nanosecond pulses to internal organs. The first is the percutaneous, bipolar, single-needle electrode designed to penetrate thyroid nodules and deliver energy there to initiate regulated cell death and reduce the volume of the nodule. Electrode placement and measurement of the reduction in nodule volume are accomplished with the aid of ultrasound imaging. The first treatments of human thyroid nodules with this applicator have shown promising volume reductions in the treated regions and this applicator will be described in the next issue of *Bioelectricity*. In addition to the percutaneous electrode, catheters are being developed, which can be guided to internal organs through blood vessels or endoscopes for energy delivery guided by various imaging modalities.

### Clinical trials using nanosecond pulsed electric fields

#### Benign skin lesions

Several clinical trials have demonstrated 80–99% efficacy in the elimination of benign skin lesions and this was the first Food and Drug Administration’s clearance for the CellFX™ System (Pulse Biosciences) applying NPS therapy in 2022. The first trial enrolled 58 subjects and treated 3 seborrheic keratoses on each.^9^ Around 82% of the treated lesions were rated clear or mostly clear by the assessing physician. The next clinical trial treated 222 sebaceous gland hyperplasia lesions on 72 subjects with 99.6% rated clear or mostly clear.^10^ A third application was the treatment of nongenital, cutaneous warts.^25^ Approximately, 75.3% of the common verrucae treated with NPS therapy were completely clear by 60 days following the last treatment and did not recur within the 120-day observation period. The most common treatment for warts uses liquid nitrogen and generally has a much higher recurrence rate. The most recent clinical trial treated acne vulgaris of the back on 17 subjects and showed an average reduction of acne lesions of 82% by 90 days after the last procedure.^11^ In summary, NPS is extremely effective, clearing every type of skin lesion tested in clinical trials thus far, and does so without any scarring or lasting pigment changes.

#### Basal cell carcinoma

The first oncology clinical trial using NPS therapy treated 10 basal cell carcinomas (BCC) on only three subjects.^3^ Seven of the treated lesions were completely free of basaloid cells when biopsied, but 2 of the 7 exhibited seborrheic keratosis in the absence of basaloid cells. One of the 10 treated lesions recurred by week 10 and, histologically, had the appearance of squamous cell carcinoma. No scar was visible on the healed sites of any of the successfully ablated lesions.

More recently, a larger trial treated 37 BCC lesions on 30 subjects, and 92% showed complete histological clearance of BCC.^26^ Histological analysis of the three cases where residual BCC was noted indicated that full energy coverage was not achieved. This study concluded that these nanosecond pulses were safe and effective for clearing both nodular and superficial BCC lesions. This success treating the deeper, nodular BCCs is noteworthy because they are resistant to treatment with other modalities.

#### Hepatocellular carcinoma

This first clinical study to evaluate the efficacy and safety of NPS for the treatment of hepatocellular carcinoma (HCC) began in 2020 in four academic medical centers in China and treated 192 patients with HCC (ClinicalTrials.gov NCT04309747).^4^ These were high-risk locations where thermal ablation was contraindicated. The applicator was composed of 2–16 parallel 19-gauge percutaneous needles applying 800 pulses of 300 ns duration and 15–30 kV/cm amplitude. The ablation zone was imaged using contrast-enhanced ultrasound, and additional cycles of energy deposition were performed to generate a margin of at least 0.5 cm around the tumor. Follow-up MR images at 16 months post-treatment revealed shrinkage of the ablation zone with 87% complete ablation, and no collateral damage to the main bile duct or hepatic vascular structure was encountered. The 87% efficacy of this first large clinical trial conducted by several different medical personnel is quite impressive and indicates that nanosecond pulsed electric fields (nsPEF) provide an effective new modality for cancer treatment.

### Future applications of nsPEF

These relatively high rates of efficacy in treating tumors that can be imaged and treated accurately point out the importance of electrode placement. The success of future applications clearly depends on the complete coverage of the tumor cells with the NPS energy. How can internal tumors be encompassed by electrode applicators? One approach for imaging inside of the body is the use of endoscopes with cameras and ultrasound transducers at their ends, which can enter the body through the openings of the throat or anus and image tumors located close to these passageways. Endoscopic ultrasound (EUS) has been used for decades to explore these internal passageways. With the addition of fine needle aspiration, EUS has been used to detect and treat many gastrointestinal luminal cancers.^27^ It has also been used to treat pancreatic disease by implementing RFA and IRE.^28^ This was done using applicator electrodes that fit within the 3 mm working channel in the endoscope and later expand to encompass the tumor imaged with the ultrasound transducer. EUS provides the highest image resolution among all the imaging modalities for pancreatic cancer because the ultrasound transducer is positioned against the inner wall of the duodenum on which the pancreas is tightly bound only millimeters away. Once EUS detects the tumor location, extending the applicator electrode out of the channel impales the tumor with the central needle and extending additional electrode arms can encompass a large region of the tumor. By repeating this process the entire tumor can be imaged and treated. Unlike surgical approaches, EUS is minimally invasive and can often be done as an outpatient procedure with much less risk of complications.

## Conclusions

NPS therapy has been very effective in clearing murine tumors as well as human basal cell carcinoma and hepatocellular carcinoma. However, many more clinical trials will be required to satisfy the regulatory agencies of its long-term safety and efficacy in oncology. The ability to stimulate the immune system, in addition to clearing the primary tumor, gives this new modality a promising future in oncology therapy.
